# Systemic Trans- and Postoperative Evaluations of Patients Undergoing Dental Implant Surgery

**DOI:** 10.6061/clinics/2016(03)07

**Published:** 2016-03

**Authors:** Marcelo Coelho Goiato, Joel Ferreira Santiago Junior, Eduardo Piza Pellizzer, Amália Moreno, Luiz Marcelo Ribeiro Villa, Stefan Fiuza de Carvalho Dekon, Paulo Sérgio Perri de Carvalho, Daniela Micheline dos Santos

**Affiliations:** Universidade Estadual Paulista (UNESP), Faculdade de Odontologia Pública; I Departamento de Odontologia, Material e Prótese Dentária; II Departamento de Cirurgia e Clínica Integrada, Araçatuba/, SP, Brazil

**Keywords:** Dental Implants, Anxiety, Alveolar Bone, Pain Perception

## Abstract

**OBJECTIVE:**

The aims of this study were to examine the trans- and postoperative systemic characteristics of patients undergoing dental implant surgery and to investigate the relationship between pre- and post- surgery anxiety levels.

**MATERIAL AND METHODS:**

Thirty-nine patients were analyzed in 3 call centers to determine anxiety levels, pain levels, and preoperative and postoperative histories using the State–Trait (STAI) questionnaire.

**RESULTS:**

A total of 93 dental implants were installed, with a success rate of 100%. The most frequently reported systemic disease was hypertension. There was a significantly higher rate of effective clamping (torque) to the mandibular bone than to the maxillary bone. The association between postoperative surgical complications and longer operative time was not significant, but there was a significant correlation between the alteration of mouth opening and daily routine activities and a significant decrease in anxiety levels between the day of surgery and the postoperative time point (*p*=0.006).

**CONCLUSION:**

A longer surgical time was associated with surgery-related complications and with a higher anxiety index on the preoperative evaluation.

## INTRODUCTION

Dental implantation has highly predictable and successful outcomes in modern times 1. The patient history and clinical examination stages are greatly important in identifying factors that may affect the surgical and rehabilitation stages of implantation.

Implantation surgeries with minimal trauma avoid stress and excessive bleeding 2. Additionally, a patient who attends a clinic to undergo dental implant surgery has several expectations with regard to treatment, such as fear of pain during the procedure 3. The placement of implants can generate minimal to moderate stress and proper management of anxiety may be related to the reduction of pain 3.

Thus, the aim of this study was to conduct pre-, trans- and post- operative monitoring of patients who received osseointegrated implants to assess systemic conditions, including the list of installed implants, mean duration of surgery, bone quality, implant clamping method, anxiety levels before and after surgery, and complications during and after surgery. The hypothesis of this study was that the most relevant patient anxiety levels would be observed prior to dental implant surgery.

## MATERIALS AND METHODS

### Subjects

This study followed the protocol of a similar experiment conducted by another group 4. This experiment complied with ethical principles, including those in the World Medical Association Declaration of Helsinki. This study was performed in accordance with the recommendations of the Committee on Ethics in Human Research (FOA –UNESP 2014/03390). Questionnaires were conducted with the understanding and written consent of each patient. A total of 39 patients were selected at 3 dental care centers (Center for Teaching and Training, Paulista Association of Dental Surgeons and UNESP - Univ Estadual Paulista). All patients sought treatment for rehabilitation from dental implantation and were registered in a sorting queue. The initial planning of each case was performed after the initial clinical examination and medical examination. When indicated based on the situation, a tentative surgery date was scheduled for the installation of an osseointegrated implant.

The inclusion criteria for patient selection were as follows: 1 patients who required the installation of dental implants; 2 patients who did not previously require or undergo bone grafting surgery; 3 patients classified as controllable ASA I or ASA II; and 4 patients with the cognitive ability understand and answer the proposed questions. The exclusion criteria were as follows: 1 patients requiring advanced surgery, such as bone grafting or lateralization of the inferior alveolar nerve, and, 2 patients presenting with uncontrolled systemic disorders.

The selected patients received information about the proposed treatment plan and provided informed consent. The researchers collected the clinical and demographic data relevant to the study.

### Experimental design

#### Preoperative

The patients selected for osseointegrated implant installation surgery underwent an anamnesis (to collect a medical history and demographic data) and a surgical planning session with a prosthetist. Reverse planning was used to schedule the surgery with the responsible surgeon at each center.

Prior to surgery, one of the researchers accompanied the preoperatively selected patients to a specific center. The questionnaires (State-Trait (STAI) questionnaire) were completed 30 minutes before performing the surgery to obtain a complete anamnesis and pain scale data (0 = no pain, 10 = worst pain).

### Transoperative

During the surgical procedure, the same researcher attended the entire transoperative step to obtain data concerning the following parameters: the profile of the patient at the moment of surgery; bone type; installation torque; amount/type of anesthetic; mean operative time for each surgeon; transoperative complications; suture stage (suture thread and postoperative medicines) and general guidelines. Additionally, data concerning the implants, such as the length, diameter and brand of the installed implant, the implantation region and the bone quality, were collected. The bone quality classification was recorded by a trained surgeon during the surgical step following the classification of Lekholm & Zarb 1985^5^ (I to IV).

### Surgical technique

The surgical stage involved the following procedure. The surgeon initially performed skin disinfection using PVP (1% active iodine). Then, the patients were administered 0.12% chlorhexidine mouthwash for 1 minute. Local infiltration anesthesia with an anesthetic together with a vasoconstrictor was applied. When necessary, after the incision and syndesmotomy, the surgical bed was prepared by milling accompanied by abundant irrigation with saline solution. Finally, an osseointegrated implant was installed for every given situation. The vast majority of the installed implants were external hexagons; therefore, the implants were installed at the bone level. After completing the surgical stage, the incised regions were sutured and medications were prescribed using the following protocol: antibiotic, amoxicillin (500 mg-1 gram 1 h before surgery followed postoperatively one 500-mg tablet every 8 hours for 7 days 6); anti-inflammatory agent, nimesulide (one 100-mg tablet 1 hour before surgery, followed postoperatively one 150-mg tablet every 12 hours for 3 days); and analgesic, acetaminophen one 750-mg tablet every 6 hours for 3 days if pain is present). For patients who were allergic to penicillin, azithromycin (one 500-mg tablet) was administered 1 h prior to the surgical procedure, followed by administration of one tablet per day for 5 days.

### Postoperative

After completing the surgical stage, the patients were followed up by the same researcher within 14 days of the procedure; during this period, the sutures were removed. At this stage, the patients were analyzed for changes in daily activities such as (a) chewing, (b) extended mouth opening, (c) communication, (d) sleep, (e) work/school activities, (f) daily routines, (g) social life, and (h) favorite activities. Additionally, each patient was consulted 3 days after the surgical procedure to evaluate the symptoms of swelling, bruising, bleeding, nausea, bad taste/bad breath, and pain. When necessary, these patients were called for an immediate postsurgical evaluation.

All patients were analyzed seven days postoperatively to determine the pain scale, the presence of impacted food and the pattern of healing. Additionally, the patients completed the STAI questionnaire on the day of surgery (time zero) and at the seven day follow-up visit time of follow-up during suture removal.

### STAI questionnaire

An analysis of anxiety was conducted for each patient using the STAI questionnaire. In the STAI-State section, the participant describes how he feels at the moment by responding to 20 items as follows: 1) absolutely not; 2) slightly; 3) somewhat; or 4) very much. In the STAI-Trait section, the participants generally describe their psychological state at that moment by responding to 20 questions with one of four answers: a) rarely; b) sometimes; c) frequently; or d) almost always.

### Statistical analysis

A descriptive statistical analysis (including frequency distribution and percentages) was performed for the demographic, medical history and installed implant data both transoperatively and postoperatively. The STAI questionnaires were used to analyze the level of anxiety of the patients and were applied in a standard manner both before and 7 days after surgery. The recent literature suggests that this questionnaire is a suitable standard to assess presurgical anxiety 7. The Portuguese version of the STAI questionnaire has been validated 8. The STAI scores are generally presented in a range from 20 to 80. In the Brazilian population, STAI scores higher than 49 indicate high anxiety and scores less than 33 indicate low anxiety, regardless of gender. Additionally, we used the Kruskal-Wallis (one-way) test and Dunn's method to determine the factors associated with effective implant clamping (>40 N) to the maxilla or the mandible. The Kruskal-Wallis test and Dunn's method were used to assess the relationship between the surgical time and surgical complications. We used the Spearman correlation test to assess the following correlations (strong: *p*≥0.8): mouth opening *vs*. daily routine activities, communication *vs*. daily routine activities, communication *vs*. social life, sleep *vs*. favorite activities, and work *vs*. favorite activities.

## RESULTS

### Patient demographics

The demographic data for the patients included in the sample are provided in Table 1. The majority of the patients were women (60%), and the mean patient age was 50.66 years. Regarding the systemic characteristics, hypertension (n=6) was the most widespread systemic disease, followed by fibromyalgia and diabetes (n=3). Regarding the presence of risk factors, 6 patients reported consistent consumption of alcoholic drinks and 3 reported the use of cigarettes (1 or 2 cigarettes/day, 1 pack/day and 2 packs/day).

### Implants and implantation region

**Figure 1:**
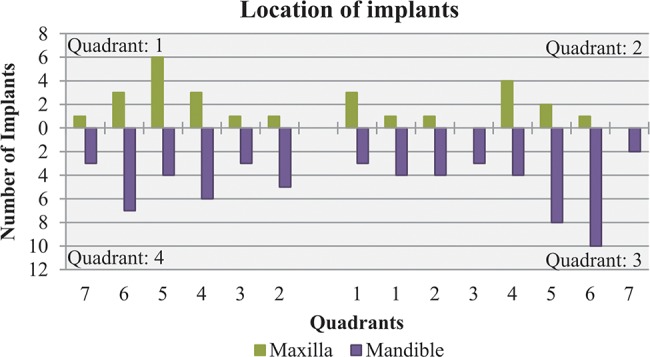
Number of implants installed in each quadrant (1 to 4): maxilla: 27 implants; mandible: 66 implants; total of 93 implants.

**Figure 2:**
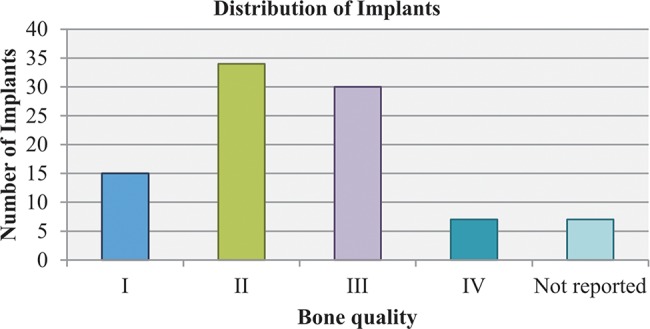
Number of implants installed according to bone quality: bone type I (n=15); bone type II (n=34); bone type III (n=30); bone type IV (n=7); and not reported (n=7).

Regarding the implant surgery data, 93 implants, including 91 external hexagons and 2 internal hexagons, were installed. The smallest diameter installed was 2.5 mm in the lateral region, the shortest length was 8.5 mm in the regions proximal to the anatomical boundaries, and the most frequently used size was 4 x 10 mm, as shown in Table 2. The sites with the highest frequency of implant installation were 36 (n=10), 35 (n=8) and 46 (n=7), as shown in Figure 1. Most common bone quality at the installation sites of the implants was bone type II (n=34), as shown in Figure 2.

### Transoperative

**Figure 3:**
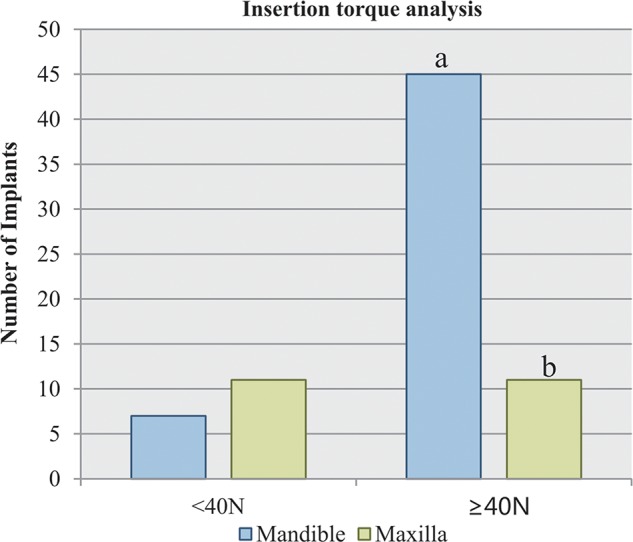
Correlation of the number of implants installed into the maxilla and the mandible with a clamping force (torque) <40 N or ≥40 N. (a, b: *p*<0.001).

The clinical and psychological statuses were assessed by the investigator prior to surgery. After inquiring about the patients' mental state, 25 patients were defined as “quiet” and 13 were defined as “anxious”; the mental state was not reported for one patient. In 38 patients, it was possible to measure the level of torque using a mechanical torque meter (classified as ≥40 N or <40 N) for the 74 implants installed. We observed that implants placed in the mandible were more effectively clamped (torque) than implants placed in the maxilla (*p*<0.001), as shown in Figure 3.

**Figure 4:**
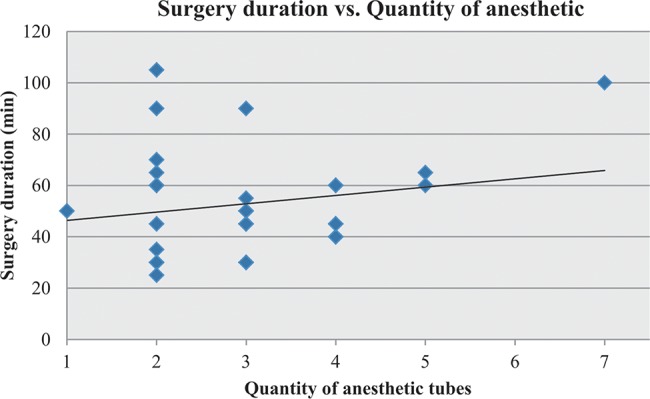
Correlation of the amount of anesthetic used with the surgical time.

**Figure 5:**
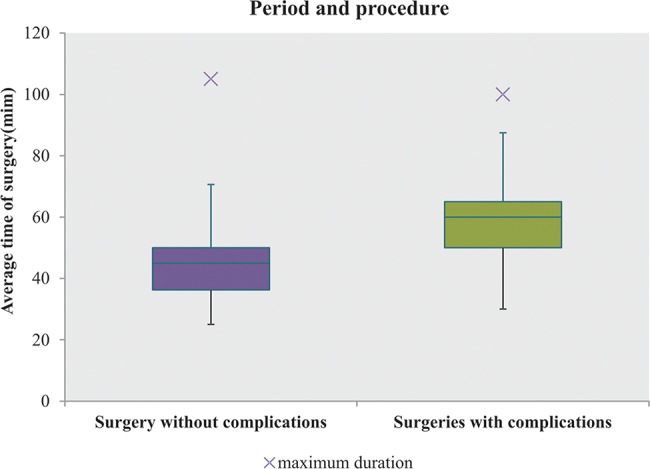
Box-plot graph showing the correlation of the average procedure time with normal surgery (uncomplicated, n=22) and surgery with complications (n=13) (*p*=0.028).

The mean amount of anesthetic used for surgery was 3.4 tubes/surgery (standard deviation (SD): 1.9). The average time of each surgery measured from the start time T_0_ (the time when the patient sat in the chair) to T_f_ (when the patient left following the procedure) was 56 minutes (SD: 26.2 min). However, 86.48% of the 35 examined patients required up to 4 vials of anesthetic, as shown in Figure 4. Dunn's post hoc test showed that surgeries with complications took a longer average time than normal surgeries (*p*=0.028), as shown in Figure 5.

### Postoperative

The largest change related to daily activities was reported for chewing food and restriction of social life. Changes in sleep and favorite activities occurred at a lower intensity. With respect to the main postoperative symptoms, the patients primarily reported swelling (slightly, n=23), and bad taste/bad breath after surgery (slightly, n=7) (Table 3). A total of 23 patients reported that they felt pain between 1 and 7 on the pain scale (VAS), with a mean of 4.6.

### Questionnaires

**Figure 6:**
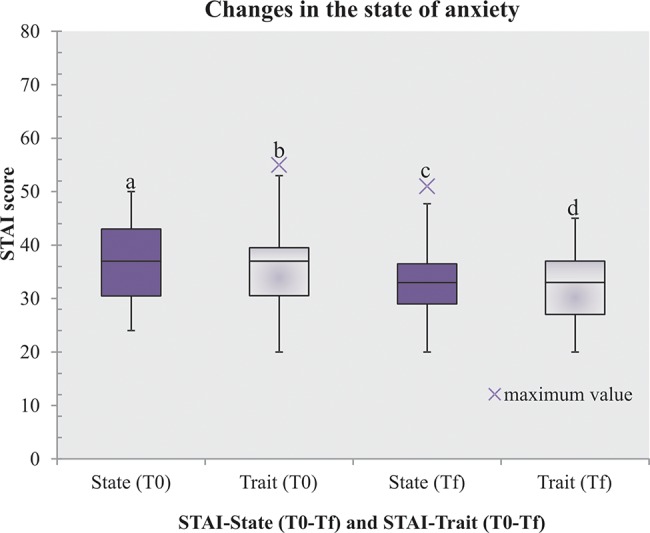
Box-plot graph of the STAI-State/Trait subscores (initial and final). Statistically significant results: a, c (*p*=0.006) and b, d (*p*=0.029).

The average STAI-State subscore observed on the first application of the questionnaire before surgery (T0) was 37.12 (SD=8.1). On the second application after 7 days (Tf), the average subscore was 33.35 (SD: 6.19). This reduction was statistically significant (t test, *p*=0.006, two-tailed test with alpha=0.808 and one-tailed test with alpha=0.887). The mean STAI-Trait subscore observed on the first application (T0) was 35.58 (SD=7.17). On the second application (Tf), the observed mean subscore was 32.33 (SD=6.07). This reduction was statistically significant (Wilcoxon, *p*=0.029, Z=-2.187), as shown in Figure 6.

**Figure 7:**
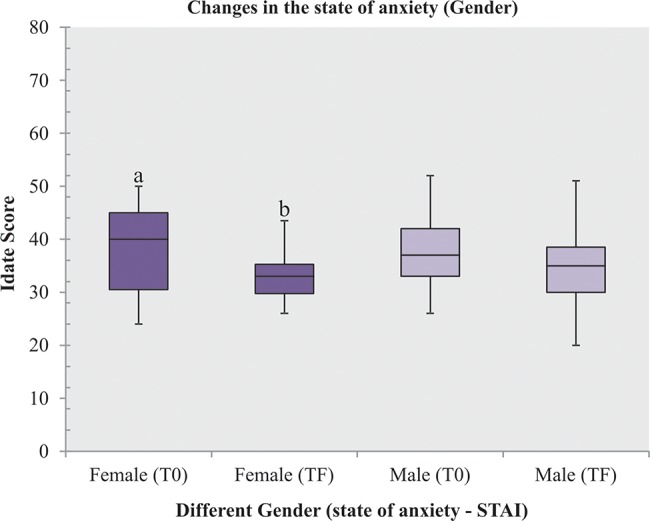
STAI-State subscores among females and males (initial and final time points) (*p*=0.01).

**Figure 8:**
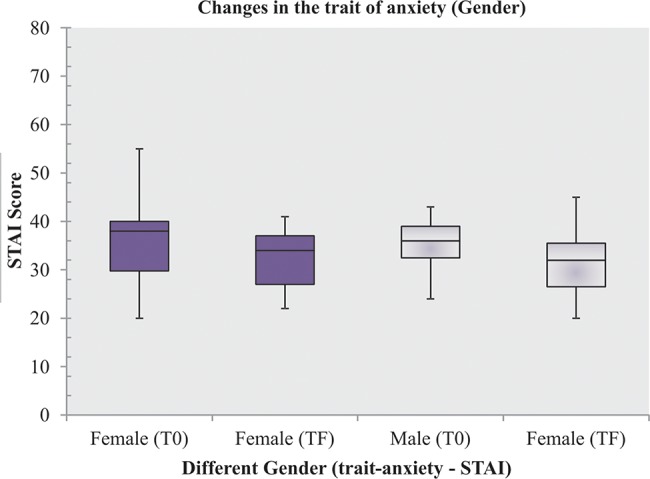
Box-plot graph of STAI-Trait subscores among females and males (initial and final time points).

When the population was classified according to gender, men had an average initial STAI-State subscore of 37.8 (SD=5.7) and this subscore was reduced to 34.5 (SD=6.8) at follow-up. Among women, a decrease from 37.7 to 33.0 was observed; based on analysis of the t test results (α=0.848), this reduction was statistically significant (*p*=0.01), as shown in Figure 7. On the STAI-Trait section, men had an average initial subscore of 34.8 and this subscore was reduced to 31.6. Finally, women showed a reduction from 36.0 (SD=8.0) to 32.7 (SD=5.6), as shown in Figure 8.

We chose to perform an analysis between the “before” and “after” scores on the STAI-State subscale using the Wilcoxon test. Patients were more secure and confident after surgery and had become more calm (*p*=0.001), more secure (*p*=0.001), more willing (*p*=0.034), more rested (*p*=0.031), less anxious (*p*=0.009) and more “at home” in the situation (*p*=0.036). Thus, the patients demonstrated greater tranquility in the postoperative period, as shown in Table 4.

## DISCUSSION

The goal of rehabilitation with functional intent was predominant. In fact, patients generally seek rehabilitation from osseointegrated implants, especially for the improvement of masticatory function, primarily because there was a greater association of improved chewing function with the insertion of implants into the posterior mandible and maxilla than into the anterior regions. Indeed, the literature shows high success rates for implant placement in the posterior region 9.

With respect to the systemic characteristics, hypertension was the most commonly reported disease among patients. Based on the average age of these patients, this characteristic may be related to cardiovascular risk 10. Thus, anamnesis was conducted prior to dental implant surgery and in cases of hypertension medical monitoring was conducted. According to Lee et al. 2010 11, there are no contraindications for osseointegrated implant surgery in patients with controlled systemic diseases. Habits including alcohol and cigarette use were also reported in this study. The literature is discordant regarding the success rate of dental implants in smokers. The negative effect of smoking was not confirmed in a recent meta-analysis. However, a protocol was adopted in which patients were requested to not smoke in connection with the installation of dental implants and during the osseointegration period 12.

With respect to implant installation, there was a predominance of implants with a length greater than 8.5 mm and this length enabled better clamping and primary implant stability as demonstrated in the literature 13. Additionally, the types of bone most commonly present were type II and type III. Based on the literature, there is a greater propensity of successful implant installation in these regions compared to implants into bone with a low density 14. More effective implant clamping was observed in the mandibular region, possibly due to the higher bone quality in this region. A recent systematic review revealed that primary implant stability and that the expertise of the surgeons may be important for the outcome of implants subjected to immediate loading; additionally, high bone tissue quality and using longer implants are important predictive factors of outcome 15.

The average time of each surgery analyzed was 26 minutes. However, we must consider that the surgeries at two centers were performed by apprentice surgeons and the surgical time may have been high due to this situation. The main complications were lipothymia, pain in mouth opening and pain during surgery that was resolved promptly by the support staff. These complications may be related to the emotional state (lipothymia) or the surgical time (pain). One of the most important aspects related to the learning curve of apprentice surgeons is that there is difficulty in clamping the implants under certain clinical conditions. Indeed, the learning curve has been associated with a lower success rate 16,17 and greater susceptibility of the installed implants 18. Our results imply that the surgical time is longer for surgeries with complications by apprentice surgeons (*p*=0.028). Recent reports in the literature showed an association between a longer surgery length and pain, the frequency of complications and, in the worst situations, increased levels of psychological distress 19.

Regarding the postoperative symptoms, we observed a correlation between mouth opening and daily routine activities, communication and daily routine activities, social life and communication, sleep and favorite activities and work and favorite activities. However, these correlations were only analyzed in a few patients. The literature suggests that some symptoms of and limitations to daily activity (mild) are to be expected in the first 3 days after implant placement 3.

The postoperative symptom most commonly reported by the patients was swelling. This result is in accordance with the literature reporting this situation in dental implant surgeries Hashem et al. 2006 3 and is possibly associated with the modification of daily activities. Pain after surgery was scored lower than 5 (0-10) by 58% of the patients. This finding is in agreement with the recent literature indicating reduced levels of pain for the installation of dental implants 20. However, this criterion may have limitations. Kim et al. 2013 20 reported that the sensation of pain could occur due to the memory of pain during local anesthesia and/or noise in the region.

Regarding the STAI-State and STAI-Trait subscales, we observed a significant reduction in scores after the surgical step (7 days). These data agree with the recent literature showing a higher rate of anxiety prior to dental implant surgery and an association of preoperative anxiety with postoperative pain 20. Furthermore, this study confirmed earlier reports that implantation surgery caused higher levels of anxiety 3, and this finding was similar to the results for oral surgeries 21,22,23.

Females had a significantly further reduction in STAI-State subscores than males. However, no such difference was observed for the STAI-Trait subscale. This discrepancy possibly occurred because the STAI-Trait section included an analysis of personality and tended to remain more stable 3. Other studies showed a greater propensity of increased anxiety among women than among men 19,24,25; however, these results should be verified in larger samples.

Recently, Enkling et al. 2013 26 conducted a follow-up study in patients classified with dental therapy phobia who received dental implants and were followed for 2-4 years. Despite the higher rate of anxiety in these patients, the authors emphasized the importance of behavior and motivation offered by the professional. Thus, the degree of learning and control techniques provided to the patient prior to the dental implant surgery is important.

Dental implant surgery has been identified as a factor that can increase the levels of stress and anxiety in patients 21. Therefore, this step should be based on adequate analgesia and management of patient anxiety, as these strategies result in a reduction in their pain experience and anxiety levels during rehabilitation treatment.

In conclusion, implants installed into the mandible showed more effective clamping than implants placed into the maxilla. A longer surgical time was associated with more frequent surgical complications.

Dental implant surgeries altered daily routine, favorite and social life activities among the analyzed patients at the 7-day time point. The analyzed patients reported higher anxiety scores during the preoperative period than during the postoperative period (7 days), especially among females.

## AUTHOR CONTRIBUTIONS

Goiato MC and Santos DM conceived and designed the study/review/case series and were also responsible for the drafting of the manuscript, critical revision, final approval and are guarantors of the manuscript. Santiago Jr JF, Carvalho PSP, Moreno A, and Villa LM were responsible for the acquisition of laboratory data or clinical/literature search results, drafting and/or critical revision of the manuscript, final approval and are guarantors of the manuscript. Pellizzer EP and Dekon SF were responsible for the analysis and interpretation of the collected data, drafting and/or critical revision of the manuscript, final approval and guarantors of the manuscript.

## Figures and Tables

**Table 1:** Characteristics of the patients according to the clinical and demographic data (n=39).

Variables	Total (%)
Gender	
Female	24 (60%)
Male	15 (40%)
Overall average age (years)	52.38
Average age of females	50.66
Average age of males	55.13
Marital status	
Single	4 (10.3%)
Married	25 (64.1%)
Divorced	5 (12.8%)
Widowed	1 (2.5%)
Not declared	4 (10.3%)
Family income	
<5 times the minimum salary in Brazil	11 (28.2%)
≥5 times the minimum salary in Brazil	7 (18%)
Not declared	21 (53.8%)
Main complaints	
Functional	34 (87.2%)
Aesthetic	5 (12.8%)

**Table 2:** Implant characteristics (n=93).

Width/Length	8.5 mm	10 mm	11.5 mm	13 mm	15 mm	Total
**2.5 mm**	0	1	0	0	0	1
**3.75 mm**	0	0	10	11	0	21
**4 mm**	10	33	15	11	1	70
**5 mm**	0	0	1	0	0	1
**Total**	10	34	26	22	1	**93**

**Table 3:** Main postoperative symptoms (n=39).

Variable/Score	A lot	A little	No change
Swelling	1	23	15
Hematoma	1	4	34
Hemorrhage	0	6	33
Nausea	1	3	35
Bad taste/Bad breath	3	7	29
Diet impacted	1	8	30
Dehiscence	0	4	35

**Table 4:** Results of the STAI-State subscale and frequency of the responses to each STAI item (n=39).

Question	Before surgery				7 days after surgery				*p*-value
STAI-State	Absolutely not	Slightly	Somewhat	A great deal	Absolutely not	Slightly	Somewhat	A great deal	
1. Calm	1(2.5)	13 (33.3)	14(35.8)	11(28.2)	0	1(2.5)	16(41.0)	22(56.4)	*p≤*0.001*
2. Secure	1(2.5)	8(20.5)	19 (48.7)	11(28.2)	0	1(2.5)	14(35.8)	24(61.5)	*p=*0.001*
3. Tense	11(28.2)	20(51.2)	8 (20.5)	0	20(51.28)	14(35.8)	2(5.0)	3(7.5%)	*p=*0.123
4. Sorry	20(51.2)	15(38.4)	3(7.5)	1(2.5)	20(51.2)	15(38.4)	2(5.0)	2(5.0)	*p=*0.869
5. Willing	1(2.5)	11(28.2)	15(38.4)	12(30.7)	1 (2.5)	1(2.5)	20(51.28)	17(43.58)	*p=*0.034*
6. Disturbed	21(53.8)	12(30.7)	4(10)	2(5)	19(48.7)	16(41)	2(5)	2(5)	*p=*0.860
7. Worried about misfortunes	15(38.4)	18(46.1)	2(5)	4(10)	20(51.2)	16(41)	3(7.5)	0	*p=*0.131
8. Rested	3(7.5)	10(25.6)	17(43.58)	9(23.0)	2(5)	4(10)	16(41.0)	17(43.5)	*p=*0.031*
9. Anxious	11(28.2)	14(35.8)	10(33.3)	4(10.0)	15(38.4)	21(53.8)	3(7.6)	0	*p=*0.009*
10. At home	2(5.1)	13(33.3)	17(43.5)	7(17.9)	1(2.5)	9(23.0)	14(35.8)	15(38.4)	*p*=0.036*
11. Confident	1(2.5)	7(17.9)	19(48.7)	12(28.2)	0	5(12.8)	15(38.4)	19(48.7)	*p*=0.071
12. Nervous	14(35.8)	18(46.1)	6(15.3)	1(2.5)	15(38.4)	20(51.2)	3(7.6)	1(2.5)	*p*=0.503
13. Agitated	16(41.0)	16(41.0)	6(15.3)	1(2.5)	16(41.0)	20(51.2)	3(7.6))	0	*p*=0.470
14. Bag of upset	21(5.3)	13(33.3)	3(7.6)	2(5.1)	18(46.1)	19(48.7)	1(2.5)	1(2.5)	*p*=0.912
15. Relaxed	2(5.1)	12(30.7)	16(41.0)	9(23.0)	4(10.2)	17(43.5)	13(33.3)	5(12.8)	*p*=0.097
16. Satisfied	0	7(17.9)	18(46.1)	14(35.8)	1(2.5)	5(12.8)	14(35.8)	19(48.7)	*p*=0.482
17. Preoccupied	14(35.8)	21(53.8)	3(7.6)	1(2.5)	13(33.3)	25(64.1)	1(2.5)	0	*p*=0.548
18. Very confused	22(56.4)	15(38.4)	1(2.5)	1(2.5)	16(41.0)	22(56.4)	1(2.5)	0	*p*=0.421
19. Happy	2(5.1)	7(17.9)	14(35.8)	16(41.0)	0	2(5.1)	15(38.4)	22(56.4)	*p*=0.019
20. Well	2(5.1))	3(7.6)	12(30.7)	22(56.4)	0	1(2.5)	12(30.7)	26(66.6)	*p*=0.108

STAI-State, State subscale of the self-administered questionnaire “State-Trait Anxiety Inventory” (STAI). Values within parentheses are percentages. The Wilcoxon test was performed. * Significant values, *p*<0.05.
